# Prognostic Value of Inflammatory Burden Index in Advanced Gastric Cancer Patients Undergoing Multimodal Treatment

**DOI:** 10.3390/cancers16040828

**Published:** 2024-02-18

**Authors:** Zuzanna Pelc, Katarzyna Sędłak, Radosław Mlak, Magdalena Leśniewska, Katarzyna Mielniczek, Piotr Rola, Jacek Januszewski, Olena Zhaldak, Anna Rekowska, Katarzyna Gęca, Magdalena Skórzewska, Wojciech P. Polkowski, Timothy M. Pawlik, Karol Rawicz-Pruszyński

**Affiliations:** 1 Department of Surgical Oncology, Medical University of Lublin, 20-080 Lublin, Poland; katarzynasedlak@umlub.pl (K.S.); lesniewska.w.magdalena@gmail.com (M.L.); kasiamielniczek@gmail.com (K.M.); jacek.januszewski000@gmail.com (J.J.); zhalek0907@gmail.com (O.Z.); arekowska@icloud.com (A.R.); katarzyna.geca@umlub.pl (K.G.); magdalena.skorzewska@umlub.pl (M.S.); wojciech.polkowski@umlub.pl (W.P.P.); karol.rawicz-pruszynski@umlub.pl (K.R.-P.); 2 Department of Laboratory Diagnostics, Medical University of Lublin, 20-080 Lublin, Poland; radoslawmlak@umlub.pl; 3 Department of Surgery, The Ohio State University Wexner Medical Center and James Comprehensive Cancer Center, Columbus, OH 43210, USA; tim.pawlik@osumc.edu

**Keywords:** inflammatory burden index, gastric cancer, neoadjuvant chemotherapy, multimodal treatment, prognostic biomarkers

## Abstract

**Simple Summary:**

Inflammatory biomarkers have been widely investigated as potential predictors of prognosis among patients with gastric cancer (GC). Recently, a novel cancer biomarker, the inflammation burden index (IBI), was proposed, which is defined as the product of C-reactive protein multiplied by the neutrophil/lymphocyte ratio. The IBI comprehensively evaluates inflammatory load in cancer patients, and to date, IBI has been validated only in the Eastern population, which is known for genetic and clinicopathological differences from Western GC patients. Therefore, this study aimed to evaluate IBI as a prognostic biomarker among Central European patients undergoing multimodal treatment for GC. A low IBI was observed among patients obtaining neoadjuvant chemotherapy (NAC), and a high IBI was associated with an increased risk of postoperative complications and a higher mortality rate. IBI might help tailor treatment decision making. However, it requires further validation in a large prospective population-based study.

**Abstract:**

Since increasing evidence underlines the prominent role of systemic inflammation in carcinogenesis, the inflammation burden index (IBI) has emerged as a promising biomarker to estimate survival outcomes among cancer patients. The IBI has only been validated in Eastern gastric cancer (GC) patients; therefore, the aim of this study was to evaluate the IBI as a prognostic biomarker in Central European GC patients undergoing multimodal treatment. Ninety-three patients with histologically confirmed GC who underwent multimodal treatment between 2013 and 2021 were included. Patient recruitment started with the standardization of neoadjuvant chemotherapy (NAC). Blood samples were obtained one day prior to surgical treatment. The textbook outcome (TO) served as the measure of surgical quality, and tumor responses to NAC were evaluated according to Becker’s system tumor regression grade (TRG). A high IBI was associated with an increased risk of postoperative complications (OR 2.95, 95% CI 1.13–7.72). In multivariate analysis, a high IBI (HR = 2.56, 95% CI 1.28–5.13) and a high neutrophil-to-lymphocyte ratio (NLR, HR = 2.55, 95% CI 1.32–4.94) were associated with an increased risk of death, while NAC administration (HR = 0.40, 95% CI 0.18–0.90) and TO achievement (HR = 0.42, 95% CI 0.22–0.81) were associated with a lower risk of death. The IBI was associated with postoperative complications and mortality among GC patients undergoing multimodal treatment.

## 1. Introduction

Gastric cancer (GC) remains a significant global health burden associated with substantial morbidity and mortality [1,2]. Despite advances in diagnosis and treatment, the prognosis of patients with GC remains poor. Current guidelines propose diverse approaches to GC treatment worldwide. While National Comprehensive Cancer Network (NCCN) and European Society for Medical Oncology (ESMO) guidelines recommend multimodal treatment based on perioperative chemotherapy along with radical gastrectomy for locally advanced disease [3,4], Japanese Gastric Cancer Association (JGCA) recommendations are limited to gastrectomy followed by adjuvant systemic treatment [5]. Although the introduction of neoadjuvant chemotherapy (NAC) has partially improved survival, progress in treatment strategies is still insufficient [6]. Given the heterogeneity of GC, the aggressive nature of the disease, and the limited response to existing therapies, relevant biomarkers to assess the prognosis and multimodal treatment results of GC patients are of clinical importance.

Increasing evidence underlines the prominent role of systemic inflammation in carcinogenesis [7]. Inflammatory biomarkers have been widely investigated as potential predictors of prognosis among patients with GC. C-reactive protein (CRP), the neutrophil-to-lymphocyte ratio (NLR), the platelet-to-lymphocyte ratio (PLR), the Glasgow prognostic score (GPS), and the systemic immune-inflammation index (SII) are associated with clinical outcomes and may be promising prognostic indicators among patients with GC [8,9,10,11,12,13,14]. However, the predictive accuracy and role of inflammatory markers in treatment decision making for patients with GC require further validation [15,16].

Recently, a novel cancer biomarker was proposed [17]. The inflammation burden index (IBI) is defined as the product of CRP multiplied by NLR. Compared with other inflammatory markers, the IBI has emerged as a possible accurate biomarker to estimate survival outcomes among cancer patients. Promising results have demonstrated a correlation between the IBI and both overall survival (OS) and disease-free survival (DFS) in a prospective cohort of patients with locally advanced GC [18]. To date, the IBI has been validated only in an Eastern population of patients, which has genetic and clinicopathological differences from Western GC patients. Therefore, the current study aimed to evaluate IBI as a prognostic biomarker among Central European patients undergoing multimodal treatment for GC.

## 2. Materials and Methods

### 2.1. Study Design and Data Source 

In this retrospective observational cohort study, patients with histologically confirmed GC who underwent multimodal treatment between 2013 and 2021 were included. The initial date of patient recruitment was set because of the standardization of NAC with 5-fluorouracil and platinum derivatives, reflecting the current evidence-based clinical guidelines for GC [3]. Preoperative staging, evaluation of the patient’s general condition, and treatment plans were carried out by a multidisciplinary team. The ypTNM stage of the disease was established according to the 8th edition of the American Joint Committee on Cancer (AJCC) [19]. Patients with early-stage GC, distant metastasis, other malignancies, incomplete clinical or pathological reports, or those who underwent upfront surgery were excluded. Importantly, patients who completed at least two cycles but did not finish the full NAC regimen were not excluded. 

No patients were lost during the observation period. The study was approved by the institutional review board (KE—0254/331/2018). The study results were reported according to the Strengthening of Reporting of Observational Studies in Epidemiology (STROBE) statement [20]. 

### 2.2. Neoadjuvant Chemotherapy

All patients received treatment based on a combination of platinum and fluoropyrimidine derivatives. The preferred regimen was FLOT-4 consisting of docetaxel at 50 mg/m^2^ on day 1, oxaliplatin at 85 mg/m^2^ on day 1, leucovorin at 200 mg/m^2^ on day 1, and 5-fluorouracil at 2600 mg/m^2^ on day 1 of the cycle, repeated every 14 days [21]. Given the inclusion period, 40% of patients received an EOX regimen (50 mg/m^2^ of epirubicin and 130 mg/m^2^ of oxaliplatin on day 1, with 625 mg/m^2^ capecitabine administered twice daily on days 1–21, repeated every three weeks). After 4–5-week time intervals, patients were scheduled for surgical treatment. Patients aged ≥  75 years were selectively qualified to obtain chemotherapy due to comorbidities and potential increased risk of treatment-related complications.

### 2.3. Inflammatory Response Markers

Blood samples used for analysis were obtained one day prior to surgical treatment. IBI was calculated based on the following formula: absolute number of CRP multiplied by the NLR.

NLR was calculated as the absolute number of neutrophils divided by the absolute number of lymphocytes in the peripheral blood. Similarly, PLR was calculated as the quotient of platelets and lymphocytes, while the lymphocyte-to-monocyte ratio (LMR) was calculated as the quotient of lymphocytes and monocytes.

### 2.4. Textbook Outcome

The concept of a textbook outcome (TO), initially introduced in colorectal cancer surgery, represents a comprehensive measure that combines various surgical metrics to offer a concise and meaningful assessment of surgical quality [22]. Among patients with GC, achieving a textbook outcome is associated with improved survival and favorable treatment outcomes [23]. In the current study, TO referred to a composite of the following quality characteristics: radical resection (macro- and microscopically), adequate lymph node yield (at least 15 lymph nodes retrieved and examined), no intra- or postoperative complications, no reinterventions, non-intensive care unit hospitalization, no prolonged hospital stay, no hospital readmission, and no 30-day mortality [24]. The cutoff for a prolonged hospital stay was set at 14 days—the 75th percentile for length of stay after gastrectomy in our institution.

### 2.5. Tumor Regression Grade

Tumor response to NAC was assessed according to histopathologic regression based on Becker’s TRG system [25,26]. The regression of the primary tumor was categorized into a 4-stage grading system and evaluated as follows: grade 1 (complete response, no residual tumor), grade 2 (subtotal regression, <10% residual tumor), grade 3 (partial regression, 10–50% residual tumor), grade 4 (no regression, >50% residual tumor). Patients with TRG = 1, 2 were categorized as chemotherapy responders and with TRG = 3, 4 as non-responders.

### 2.6. Endpoints of the Study 

The study was focused on evaluating several endpoints. The primary endpoint was OS, and the secondary endpoints included postoperative complications, response to NAC according to TRG, and achieving TO. 

### 2.7. Statistical Analysis

Statistical analysis of the data was performed using the MedCalc v.15.8 software (MedCalc Software, Ostend, Belgium). To reject the null hypothesis, a *p*-value below 0.05 was used. To mitigate the risk of a type II error, we set a cutoff for beta at 0.2 to achieve 80% statistical power. Given the lack of prior research on IBI evaluation in GC patients undergoing multimodal therapy, we determined the sample size based on a study conducted by Ding et al. given the methodological resemblance [18]. The sample size calculation was conducted by comparing the percentages of patients with a 3-year survival and a primary endpoint—IBI (low or high). Considering the percentage of patients with 3-year survival in groups with high (85%) and low (100%) IBI and the ratio of sample sizes in the compared groups (1.4:1), the minimal study group was estimated as 91 patients. Given the absence of a normal data distribution (assessed by the D’Agostino–Pearson test), the median and the interquartile range or minimum–maximum range were used to present the concentration and dispersion of the data. Categorized or dichotomized variables were represented as numbers and percentages. Comparisons of IBI values, depending on demographic and clinical variables, were performed using the Mann–Whitney U test (comparisons of two independent groups) or ANOVA Kruskal–Wallis (comparisons of more than two independent groups). Receiver operating characteristic (ROC) analysis was used in the assessment of the diagnostic usefulness of the IBI value in the prediction of the occurrence of postoperative complications. The influence of demographic and clinical variables on the risk of postoperative complications was assessed based on the calculation of odds ratio (OR) and corresponding 95% confidence intervals (CIs). OS was defined as the time from the date of surgery to either the patient’s date of death (for complete data) or the last follow-up date (for censored data). In univariable survival analysis, the log-rank test was used to calculate the proportional hazard ratio (HR) with a corresponding 95% CI, and the Kaplan–Meier estimation method was used to generate survival curves. For multivariable analysis, Cox logistic regression models were used in multivariable survival analysis. Two-sided tests were used for all analyses, and statistical significance was defined as a *p*-value below 0.05.

## 3. Results

### 3.1. Patient Characteristics

Among the 93 patients who met the inclusion criteria, the median age at diagnosis was 61 (range 32–83 years); most patients were men (64.5%). According to Lauren’s classification, almost half of the patients had intestinal-type GC (49.5%); the majority of patients (84.9%) successfully completed the planned NAC cycles. The most common surgical procedure was total gastrectomy (41.9%). Postoperative complications occurred in 28% of patients. Detailed clinicodemographic characteristics are noted in Table 1.

### 3.2. Comparison of IBI Depending on Selected Demographic and Clinical Variables

A lower median IBI was observed among patients who received versus did not receive NAC (7.9 vs. 185.7, respectively; *p* = 0.0002). Additionally, a higher median IBI was observed among patients with postoperative complications (32.8 vs. 7.9; *p* = 0.0499). Detailed data comparing IBIs based on selected demographic and clinical variables are presented in Table 2.

**Figure 1:**
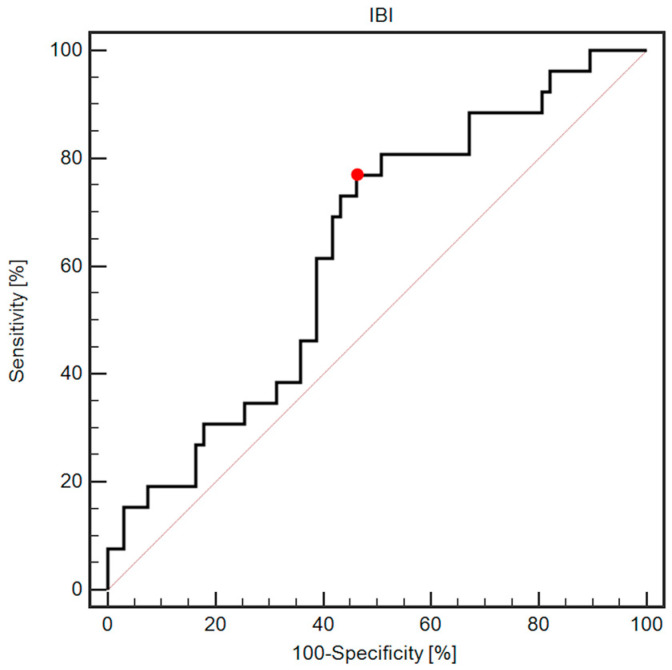
ROC curve demonstrating diagnostic usefulness of IBI assessment in predicting postoperative complications. The red dot indicates a cutoff value of 9.7.

ROC analysis revealed that the IBI (cutoff > 9.7) was associated with 76.9% sensitivity and 53.7% specificity in predicting postoperative complications (AUC = 0.63, 95% CI: 0.53–0.73; *p* = 0.0354; Figure 1).

### 3.3. Influence of Selected Demographic and Clinical Variables on the Postoperative Complications

**Figure 2:**
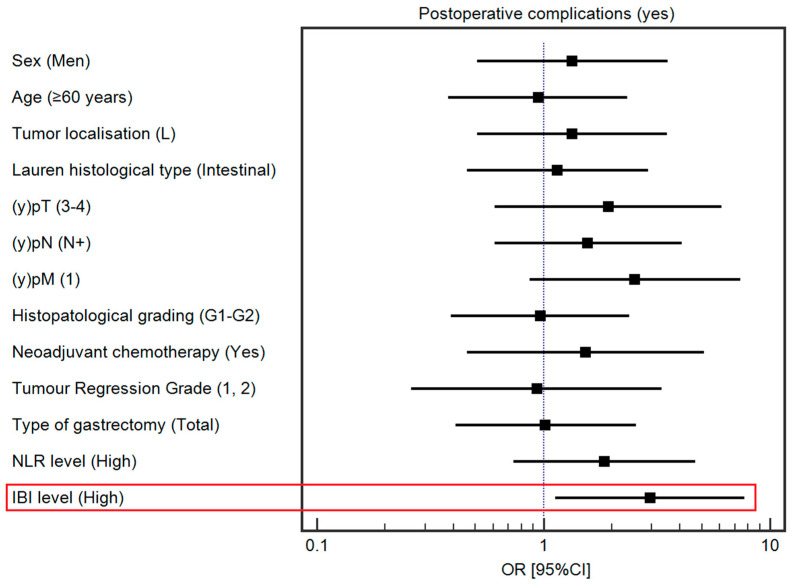
Forest plot demonstrating the influence of selected demographic and clinical variables on the risk of postoperative complications. The red square highlights statistically significant results.

After controlling for demographic and clinical variables, a high IBI was associated with a higher risk of postoperative complications (OR 2.95, 95% CI 1.13–7.72). Detailed data regarding the influence of selected demographic and clinical variables on postoperative complications are presented in Supplementary Table S1 and Figure 2.

### 3.4. Influence of Selected Demographic and Clinical Variables on Overall Survival

**Figure 3:**
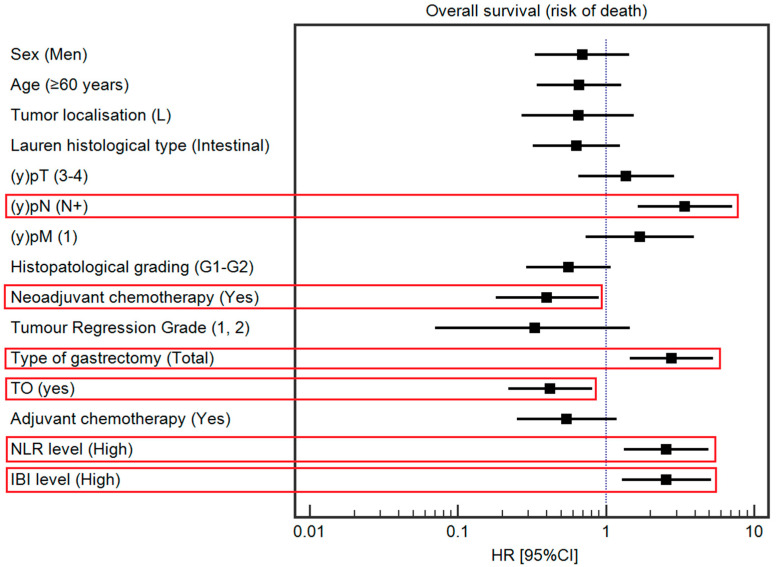
Forest plot demonstrating a multivariable analysis of the influence of selected demographic and clinical variables on the risk of death. The red square highlights statistically significant results.

**Figure 4:**
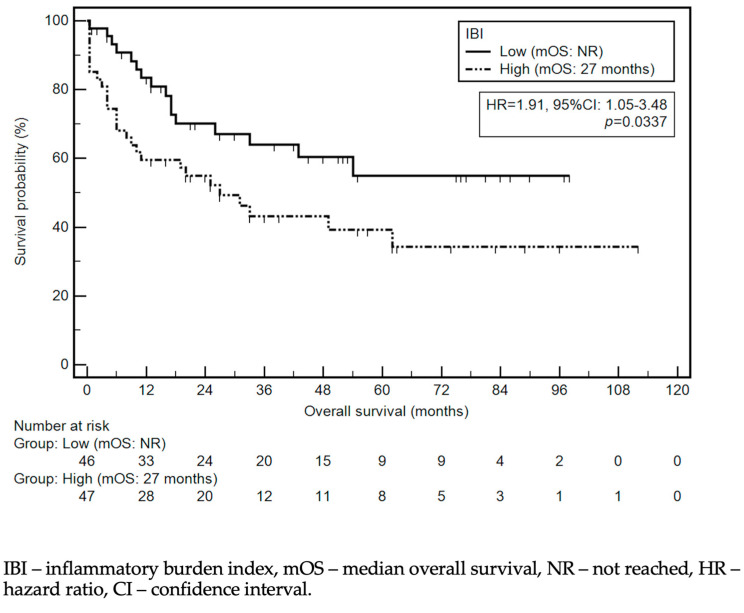
Kaplan–Meier curves demonstrating survival probability depending on IBI level.

The median OS was 49 months. Detailed data regarding the association between the selected demographic and clinical variables and OS are presented in Table 3 and Figure 3. Among the studied demographic and clinical variables, lower tumor localization (HR = 0.42, 95% Cl 0.22–0.78), Lauren’s intestinal type (HR = 0.43, 95% CI: 0.23–0.79), tumor response to chemotherapy (HR = 0.24, 95% CI: 0.10–0.55), and TO (HR = 0.51, 95% CI: 0.28–0.93) were associated with a lower risk of death. In contrast, a high IBI (HR = 1.91, 95% CI: 1.05–3.48); (y)pT4 and low-grade tumors (HR = 1.98, 95% CI: 0.82–4.77; HR = 2.03, 95% CI: 1.12–3.71, respectively); lymph node metastases (HR = 3.54, 95% CI: 1.93–6.49); and total gastrectomy (HR = 2.32, 95% CI: 1.24–4.33) were associated with a higher risk of death. The association between survival and the IBI is depicted in Figure 4.

In multivariable analysis, among studied demographic and clinical variables, NAC administration (HR = 0.40, 95% CI: 0.18–0.90) and achievement of TO (HR = 0.42, 95% CI: 0.22–0.81) were associated with a lower risk of death. On the other hand, (y)pN (HR = 3.41, 95% CI: 1.64–7.12), type of gastrectomy (HR = 2.77, 95% CI: 1.45–5.31), a high IBI (HR = 2.56, 95% CI: 1.28–5.13; Figure 3), and a high NLR (HR = 2.55, 95% CI: 1.32–4.94) were associated with a higher risk of death.

The correlation between the selected demographic and clinical variables and IBI remained insignificant, except for NLR (rho = 0.672, *p* < 0.0001), PLR (rho = 0.391, *p* = 0.0001), and LMR (rho = −0.475, *p* < 0.0001) (Supplementary Table S2).

## 4. Discussion

Inflammatory markers reflect the correlation between the tumor microenvironment and the host immune response [27]. These markers can assist in tailoring therapeutic strategies among patients with GC as indicators of tumor aggressiveness and treatment response. One of the most promising and innovative prognostic markers, the IBI, comprehensively evaluates inflammatory load in cancer patients [17]. To the best of our knowledge, this is the first study to assess the IBI as a prognostic marker in a Central European GC population undergoing multimodal treatment. Patients who received NAC demonstrated a lower IBI compared with individuals who underwent upfront surgery. Furthermore, a high IBI was the sole variable associated with an increased risk of postoperative complications. In addition, a high IBI was associated with an increased risk of death.

The IBI has emerged as a valuable tool in assessing the complexity of the inflammatory process by incorporating three essential parameters: CRP, neutrophils, and lymphocytes. Increased serum CRP is thought to be the most significant clinical parameter of acute inflammation, while neutrophils and lymphocytes are vital cellular components in the immune response [27,28,29]. Cancer patients experience dysregulation of the balance between pro- and anti-inflammatory processes, contributing to tumor growth, progression, and metastasis [30]. The first comprehensive analysis of these three parameters combined into an IBI biomarker was presented by Xie et al. in their prospective multicenter analysis of 6359 cancer patients [17]. The IBI was an independent high-risk factor associated with life functions, nutritional status, and short-term outcomes. In the current analysis, only high IBIs demonstrated an association with an increased risk of postoperative complications. Of note, NLR alone without CRP was not associated with morbidity. This finding emphasized the important role of both CRP and monitoring its values in a patient’s clinical presentation in the postoperative course. Despite significant variation in reported cutoff values, numerous studies have confirmed the potential of CRP levels as an early predictor of major postoperative complications after gastrectomy [31,32,33]. The serum-CRP peak is observed approximately 48 h after initiating an acute inflammatory response among patients with minor or no complications, and the highest diagnostic accuracy for the ratio of CRP levels in the early prediction of major postoperative complications was demonstrated from postoperative day 3 to day 2 in [34]. The inflammatory burden following gastrectomy could be modified through interventions such as preoperative steroid injections or perioperative probiotic supplementation [35,36,37]. Both procedures have demonstrated promising results in improving short-term outcomes, which could impact the proportion of patients eligible for adjuvant chemotherapy. Several ongoing studies will hopefully verify these results in a randomized setting and potentially modify clinical decision making [38].

To date, there has only been one attempt to verify the applicability of the IBI in a population of locally advanced GC patients [18]. Among 103 patients undergoing curative intent gastrectomy, 60 patients (58.25%) had a high IBI, and the remaining group was classified as IBI-low. The results demonstrated worse treatment outcomes among patients with a higher inflammatory burden. However, the available data on NAC administration demonstrated inconsistent results. In contrast, with a cohort of 84.9% of patients who underwent preoperative chemotherapy, the median IBI in the NAC group was 7.9 versus 185.7 in the group subjected to upfront surgery. A recent evaluation assessing the predictive role of changes in inflammatory and nutritional markers during the perioperative period among patients with advanced GC demonstrated that multiple cycles of NAC decreased CRP and lymphocytes and increased neutrophils [39]. Therefore, preoperative chemotherapy administration can minimize the inflammation burden with a further potential impact on the patient’s survival.

The relationship between IBI and survival among cancer patients has been a subject of previous research [17,18]. One analysis highlighted that IBI-low patients had better survival than IBI-high individuals (69.1% vs. 45.7%, respectively). In research dedicated to locally advanced GC patients, a high IBI was correlated with a lower 5-year OS and DFS compared with individuals who were IBI-low (OS: 70% vs. 791%; DFS: 50% vs. 74.4%) [18]. The differences in survival were also noted in a subgroup analysis and concerned all pathological stages. Despite the differences in treatment regimens, the results of this previous study were consistent with the current study. Along with NAC, the achievement of a TO was an independent prognostic factor in multivariable analysis. However, future research should focus on larger cohorts to provide a more comprehensive understanding of the IBI’s prognostic value in GC patients.

The results of this study should be interpreted within the context of certain limitations. This single-institution research had a retrospective design, which may limit the generalizability of the findings. A limited dataset allowed for IBI evaluation at a single time point only. Further studies should focus on continuous IBI assessment throughout the multimodal treatment of GC patients and validate the utility of IBI assessment as a predictive factor for postoperative complications and a prognostic factor in a larger cohort.

## 5. Conclusions

The IBI might be associated with postoperative complications and mortality among GC patients undergoing multimodal treatment. Further research is warranted to validate the IBI as a reliable risk stratification and treatment decision-making marker.

## Figures and Tables

**Table 1:** Patients’ characteristics.

Variable	Study Group (*n* = 93) *n* (%) or Median (Range)
**Sex**	
Men	60 (64.5%)
Women	33 (35.5%)
**Age**	61 (32–83)
**Lauren’s type**	
Intestinal	45 (49.5%)
Mixed	22 (24.2%)
Diffuse	24 (26.4%)
**Tumor localization**	
U	22 (23.66%)
M	43 (46.2%)
L	28 (30.1%)
**(y)pT**	
0	4 (4.3%)
1	7 (7.6%)
2	19 (20.4%)
3	48 (51.6%)
4	15 (16.1%)
**(y)pN**	
0	41 (44.1%)
1	14 (15.1%)
2	13 (14%)
3a	9 (9.7%)
3b	16 (17.2%)
**Grading**	
G1	5 (5.4%)
G2	42 (45.2%)
G3	46 (49.5%)
**Neoadjuvant chemotherapy**	
Yes	79 (84.9%)
No	14 (15.1%)
**No. of neoadjuvant chemotherapy cycles**	3 (2–8)
**Tumor regression grade**	
1	4 (5.2%)
2	12 (15.6%)
3	36 (46.8%)
4	25 (32.5%)
N/A: *n* = 14	
No data: *n* = 2	
**Type of gastrectomy**	
Proximal	21 (22.6%)
Distal	33 (35.5%)
Total	39 (41.9%)
**Surgical margin**	
R0	85 (91.4%)
R1	8 (8.6%)
**CCI**	20.9 (0–100)
**Postoperative complications**	
No	67 (72%)
Yes	26 (28%)
**Unplanned ICU**	
No	77 (82.8%)
Yes	16 (17.2%)
**ICU stay [days]**	2.5 (1–9)
**TO**	
No	44 (47.8%)
Yes	48 (52.2%)
No data: *n* = 1	
**Adjuvant chemotherapy**	
No	28 (35%)
Yes	52 (65%)
No data: *n* = 13	

N—number, U—upper, M—median, L—lower, ypT—post-neoadjuvant pathological tumor stage, ypN—post-neoadjuvant pathological nodal stage, CCI—comprehensive complication index, ICU—intensive care unit, TO—textbook outcome.

**Table 2:** Comparison of IBIs depending on selected demographic and clinical variables.

Variable	IBI Median [Interquartile Range]	*p*
**Sex**		
Men	10.7 [2.9–144.95]	0.319
Women	19.2 [3.2–281.1]	
**Age**		
<75 years	9.97 [2.85–181.10]	0.078
≥75 years	59.06 [20.06–848.65]	
**Tumor localization**		
U	19.2 [3.9–128.9]	0.268
M	15.3 [3–156.5]	
L	7.5 [1.7–241.7]	
**Lauren’s type**		
Intestinal	10.0 [2.3–72.1]	0.245
Mixed	8.6 [3.2–157.6]	
Diffuse	56.2 [4.6–441.9]	
**(y)pT**		
0	2.3 [11–17.3]	0.439
1a	93 [79–178.2]	
1b	140.8 [7.9–267.5]	
2	5.4 [1.6–258.3]	
3	16.2 [2.9–155.4]	
4a	18.9 [6–217.4]	
4b	256.5 [39.2–816.4]	
**(y)pN**		
0	10 [2–159.4]	0.284
1	9.3 [1.5–68.4]	
2	22 [3.1–416.2]	
3a	6.6 [1.7–97.9]	
3b	55.4 [13.1–242.8]	
**Histopathological grading**		
G1	31.5 [4.3–261.1]	0.963
G2	19.6 [2.8–182]	
G3	10.7 [3.3–153.1]	
**Neoadjuvant chemotherapy**		
Yes	7.9 [2.5–124]	0.0002 *
No	185.7 [68.5–780.7]	
**Tumor regression grade**		
1	2.3 [1.5–68.4]	0.509
2	7.1 [1.4–189.1]	
3	6.2 [2.6–70]	
4	10.5 [3.6–121.8]	
**Tumor regression grade**		
1 or 2	4.6 [1.4–161.4]	0.474
3 or 4	7.9 [2.8–72.1]	
**Postoperative complications**		
No	7.9 [2.5–171.9]	0.0499 *
Yes	32.8 [10–287.3]	
**Unplanned ICU**		
No	9.7 [2.7–184.8]	0.199
Yes	28.7 [10.4–212.3]	

IBI—inflammatory burden index, U—upper, M—median, L—lower, ypT—post-neoadjuvant pathological tumor stage, ypN—post-neoadjuvant pathological nodal stage, ICU—intensive care unit, *—statistical significance.

**Table 3:** Influence of selected demographic and clinical variables on overall survival.

Variable	mOS (Months)	Univariable		Multivariable	
		HR [95% CI]	*p*	HR [95% CI]	*p*
**Sex**					
Women	49	0.90 [0.48–1.68]	0.7288	0.69 [0.33–1.44]	0.3292
Men	NR				
**Age**					
<75 years	43	0.70 [0.29–1.73]	0.4946	0.69 [0.25–1.94]	0.4853
≥75 years	NR				
**Tumor localization**					
U, M	33	0.42 [0.22–0.78]	0.0189 *	0.65 [0.27–1.54]	0.3274
L	NR				
**Lauren histological type**					
Intestinal	NR	0.43 [0.23–0.79]	0.0057 *	0.63 [0.32–1.24]	0.1848
Diffuse/Mixed	25				
**(y)pT**					
0–3	54	1.98 [0.82–4.77]	0.0502 *	1.37 [0.65–2.90]	0.4098
4	17				
**(y)pN**					
N0	NR	3.54 [1.93–6.49]	0.0001 *	3.41 [1.64–7.12]	0.0011 *
N+	18				
**Histopathological grading**					
G3	NR	2.03 [1.12–3.71]	0.0193 *	0.56 [0.29–1.08]	0.0844
G1, G2	26				
**Neoadjuvant chemotherapy**					
Yes	54	0.45 [0.17–1.19]	0.0593 *	0.40 [0.18–0.90]	0.0278 *
No	8				
**Tumor regression grade**					
3, 4	NR	0.24 [0.10–0.55]	0.0304 *	0.33 [0.07–1.45]	0.1441
1, 2	43				
**Type of gastrectomy**					
Proximal, Distal	NR	2.32 [1.24–4.33]	0.0041 *	2.77 [1.45–5.31]	0.0022 *
Total	16				
**TO**					
No	31	0.51 [0.28–0.93]	0.0269 *	0.42 [0.22–0.81]	0.0094 *
Yes	NR				
**Adjuvant chemotherapy**					
No	49	0.64 [0.31–1.31]	0.1877	0.54 [0.25–1.18]	0.1246
Yes	NR				
**LMR**					
Low	NR	0.86 [0.47–2.57]	0.6157	0.82 [0.44–1.54]	0.5438
High	54				
**PLR**					
Low	62	1.46 [0.80–2.67]	0.1975	1.07 [0.55–2.06]	0.8436
High	25				
**NLR**					
Low	NR	2.58 [1.41–4.73]	0.0017 *	2.55 [1.32–4.94]	0.0056 *
High	18				
**IBI**					
Low	NR	1.91 [1.05–3.48]	0.0337 *	2.56 [1.28–5.13]	0.0083 *
High	27				

mOS—median overall survival, HR—hazard ratio, CI—confidence interval, U—upper, M—median, L—lower, ypT—post-neoadjuvant pathological tumor stage, ypN—post-neoadjuvant pathological nodal stage, TO—textbook outcome, LMR—lymphocyte/monocyte ratio, PLR—platelet/lymphocyte ratio, NLR—neutrophil/lymphocyte ratio, IBI—inflammatory burden index, *—statistical significance.

## Data Availability

Upon special request and in accordance with ethical considerations, the authors will share the research data supporting the reported results.

## Supplementary Materials

The following supporting information can be downloaded at https://www.mdpi.com/article/10.3390/cancers16040828/s1: Table S1: Influence of selected demographic and clinical variables on the risk of postoperative complications. Table S2: Assessment of the correlations between the selected demographic and clinical variables and IBI values.
