# Supplementary material for: Prognostic Value of Inflammatory Burden Index in Advanced Gastric Cancer Patients Undergoing Multimodal Treatment
Source: Cancers (Basel). 2024 Feb 18;16(4):828. doi: 10.3390/cancers16040828 (PMC10886992; doi:10.3390/cancers16040828)
Supplement: Supplementary file 1 [file cancers-16-00828-s001.zip › Supplementary Table S1.pdf]

Supplementary Table S1. Influence of Selected Demographic and Clinical Variables on the Risk of Postoperative Complications.

Variable	Postoperative complications		Univariable#	
	No	Yes	OR [95%CI]	*p*
**Sex**				
Women	25 (75.8%)	8 (24.2%)	1.34 [0.51-3.53]	0.554
Men	42 (70%)	18 (30%)		
**Age**				
<75 years	61 (73.5%)	22 (26.5%)	1.85 [0.48-7.17]	0.374
≥75 years	6 (60%)	4 (40%)		
**Tumor localisation**				
U, M	48 (73.8%)	17 (26,2%)	1.34 [0.51-3.52]	0.555
L	19 (67.9%)	9 (32.1%)		
**Lauren histological type**				
Diffuse/Mixed	34 (73.9%)	12 (26.1%)	1.15 [0.46-2.89]	0.764
Intestinal	32 (71.1%)	13 (28.9%)		
**(y)pT**				
0-2	58 (74.4%)	20 (25.6%)	1.93 [0.61-6.11]	0.261
3-4	9 (60%)	6 (40%)		
**(y)pN**				
N-	31 (77.5%)	9 (22.5%)	1.57 [0.61-4.07]	0.348
N+	35 (68.6%)	16 (31.4%)		
**Histopathological grading**				
G3	33 (71.7%)	13 (28.3%)	0.97 [0.39-2.40]	0.948
G1-G2	34 (72.3%)	13 (27.7%)		
**Neoadjuvant chemotherapy**				
No	9 (64.3%)	5 (35.7%)	1.53 [0.46-5.10]	0.485

Yes	58 (73.4%)	21 (26.6%)		
**Tumour Regression Grade**				
3, 4	45 (73.8%)	16 (26.2%)	0.94 [0.26-3.33]	0.920
1, 2	12 (75%)	4 (25%)		
**Type of gastrectomy**				
Proximal, Distal	39 (72.2%)	15 (27.8%)	1.02 [0.41-2.56]	0.963
Total	28 (71.8%)	11 (28.2%)		
**LMR**				
Low	33 (71.7%)	13 (28.3%)	0.97 [0.39-2.40]	0.9485
High	34 (72.3%)	13 (27.7%)		
**PLR**				
Low	34 (73.9%)	12 (26.1%)	1.20 [0.48-2.98]	0.6911
High	33 (70.2%)	14 (29.8%)		
**NLR**				
Low	36 (78.3%)	10 (21.7%)	1.86 [0.74-4.68]	0.189
High	31 (66%)	16 (34%)		
**IBI**				
Low	38 (82.6%)	8 (17.4%)	2.95 [1.13-7.72]	**0.0277***
High	29 (61.7%)	18 (38.3%)		

U – upper, M – median, L – lower, ypT – post-neoadjuvant pathological tumor stage, ypN - post-neoadjuvant pathological nodal stage, LMR – lymphocyte/monocyte ratio, PLR – platelet/lymphocyte ratio, NLR – neutrophil/lymphocyte ratio, IBI – inflammatory burden index
