# Supplementary material for: Prognostic Value of Inflammatory Burden Index in Advanced Gastric Cancer Patients Undergoing Multimodal Treatment
Source: Cancers (Basel). 2024 Feb 18;16(4):828. doi: 10.3390/cancers16040828 (PMC10886992; doi:10.3390/cancers16040828)
Supplement: Supplementary file 1 [file cancers-16-00828-s001.zip › Supplementary Table S2-kopia.pdf]

Supplementary Table S2. Assessment of the Correlations Between Selected Demographic and Clinical Variables and IBI Values.

**Variable**	**IBI**	
	**rho**	***p***
**Age [years]**	0.174	0.0948
**Lauren`s type**	0.149	0.1591
**(y)pT**	0.141	0.1765
**(y)pN**	0.150	0.1510
**Histopathological grading**	-0.009	0.9296
**No. of neoadjuvant chemotherapy cycles**	-0.095	0.4145
**Tumour Regression Grade**	0.117	0.3125
**CCI**	0.065	0.5383
**ICU stay [days]**	0.216	0.4219
**LMR**	-0.475	**<0.0001***
**PLR**	0.391	**0.0001***
**NLR**	0.672	**<0.0001***

ypT – post-neoadjuvant pathological tumor stage, ypN - post-neoadjuvant pathological nodal stage, LMR – lymphocyte/monocyte ratio, PLR – platelet/lymphocyte ratio, NLR – neutrophil/lymphocyte ratio, IBI – inflammatory burden index, CCI – Comprehensive Complication Index, ICU – intensive care unit, LMR – lymphocyte/monocyte ratio, PLR – platelet/lymphocyte ratio, NLR – neutrophil/lymphocyte ratio
